# Mitochondrial Echoes of First Settlement and Genetic Continuity in El Salvador

**DOI:** 10.1371/journal.pone.0006882

**Published:** 2009-09-02

**Authors:** Antonio Salas, José Lovo-Gómez, Vanesa Álvarez-Iglesias, María Cerezo, María Victoria Lareu, Vincent Macaulay, Martin B. Richards, Ángel Carracedo

**Affiliations:** 1 Unidade de Xenética, Departamento de Anatomía Patolóxica e Ciencias Forenses, and Instituto de Medicina Legal, Facultade de Medicina, Universidade de Santiago de Compostela, Galicia, Spain; 2 Laboratorio de Genética Forense, Instituto de Medicina Legal, Dr. Roberto Masferrer, Corte Suprema de Justicia, San Salvador, El Salvador; 3 Department of Statistics, University of Glasgow, Glasgow, United Kingdom; 4 Institute of Integrative and Comparative Biology, Faculty of Biological Sciences, University of Leeds, Leeds, United Kingdom; Erasmus University Medical Center Rotterdam, Netherlands

## Abstract

**Background:**

From Paleo-Indian times to recent historical episodes, the Mesoamerican isthmus played an important role in the distribution and patterns of variability all around the double American continent. However, the amount of genetic information currently available on Central American continental populations is very scarce. In order to shed light on the role of Mesoamerica in the peopling of the New World, the present study focuses on the analysis of the mtDNA variation in a population sample from El Salvador.

**Methodology/Principal Findings:**

We have carried out DNA sequencing of the entire control region of the mitochondrial DNA (mtDNA) genome in 90 individuals from El Salvador. We have also compiled more than 3,985 control region profiles from the public domain and the literature in order to carry out inter-population comparisons. The results reveal a predominant Native American component in this region: by far, the most prevalent mtDNA haplogroup in this country (at ∼90%) is A2, in contrast with other North, Meso- and South American populations. Haplogroup A2 shows a star-like phylogeny and is very diverse with a substantial proportion of mtDNAs (45%; sequence range 16090–16365) still unobserved in other American populations. Two different Bayesian approaches used to estimate admixture proportions in El Salvador shows that the majority of the mtDNAs observed come from North America. A preliminary founder analysis indicates that the settlement of El Salvador occurred about 13,400±5,200 Y.B.P.. The founder age of A2 in El Salvador is close to the overall age of A2 in America, which suggests that the colonization of this region occurred within a few thousand years of the initial expansion into the Americas.

**Conclusions/Significance:**

As a whole, the results are compatible with the hypothesis that today's A2 variability in El Salvador represents to a large extent the indigenous component of the region. Concordant with this hypothesis is also the observation of a very limited contribution from European and African women (∼5%). This implies that the Atlantic slave trade had a very small demographic impact in El Salvador in contrast to its transformation of the gene pool in neighbouring populations from the Caribbean facade.

## Introduction

El Salvador lies on the Pacific coast (without an Atlantic seaboard) and it is the smallest of the Central American countries. Most of the country rests on a fertile volcanic plateau. It is segmented by two volcanic ranges running roughly west to east, separated by broad, fertile valleys, such as that of the river Lempa. El Salvador was inhabited by Native American groups who were in part descendants of the Aztecs and Toltec of Mexico, such as the Pipil (a Nahua tribe) and the Lenca. These two Native American communities inhabited mainly the western regions, constituting about 60% of the population throughout the colonial era and into the early decades of independence [1], [2].

The development of coffee estates led to the slow but continuous dissolution of most of the communal lands of Native villages [1], [2]. Thus, the 1930 census, the last to contain the category, designated only 5.6% of the population as “Indian” – although it is not clear what criteria were used in arriving at this figure. Other independent estimates (considering religious activities, distinctive women's dress, language, and involvement in various handicrafts) placed the mid-twentieth-century Indian population at 20% (∼400,000 persons). The abandonment of Indian language and customs was hastened by political repression; most natives stopped wearing traditional dress, abandoned the Pipil language, and adopted ladino customs. By 1975 no more than ∼1% of the population wore distinctive Indian clothing or followed Indian customs. Nowadays, the official language in El Salvador is Spanish, although Nahua is still spoken among some natives.

Although the American continent has been the target of many forensic and population genetic studies, there are nevertheless many American regions, such as El Salvador, that remain genetically uncharacterized. The mtDNA molecule is commonly used in anthropological contexts because of particular features (maternal inheritance, lack of recombination and high average mutation rate) that confer great power for phylogenetic and phylogeographic inferences. Many mtDNA studies of Native Americans have, however, been limited to genotyping a handful of mtDNA coding region sites that simply distinguish the four major Native American mtDNA haplogroups, A2, B2, C1 and D1 (generally using RFLP typing); unfortunately, the information provided by these few SNPs is of limited value in forensic and population genetics.

Here we have sequenced the mtDNA control region in a sample from El Salvador in order to investigate to what extent the Native American component has survived the impact of European colonialism and the concomitant influx of African slaves to the Caribbean and Meso-America.

## Materials and Methods

### Sample collection and DNA extraction

A total of 90 saliva samples were collected from healthy unrelated individuals from El Salvador. DNA extraction was undertaken following standard phenol-chloroform protocol. DNA quantification was carried out using DyNA Quant 200 Fluorometer, Hoefer (APB, Uppsala, Sweden).

All the samples were collected anonymously by the Laboratorio de Genética Forense from the Instituto de Medicina Legal that belongs to the Corte Suprema de Justicia from San Salvador (El Salvador). Oral informed consent was required in all the cases. The study, including the oral informed consent protocol, was approved by the Ethical committee of the University of Santiago de Compostela, and it conforms to the Spanish Law for Biomedical Research (Law 14/2007- 3 of July).

### PCR and sequence analysis

We analyzed the first and second hypervariable segments (HVS-I and HVS-II) of the mtDNA genome. We performed PCR amplifications using a 2700 Thermocycler (Applied Biosystems), using PCR and sequencing primers as reported in [3]. Cycling parameters were 95°C for 1 min, followed by 36 cycles of 95°C for 10 sec, 55°C for 30 sec and 72°C for 30 sec, and followed by 10 min at 15°C. We checked amplification products on a polyacrylamide gel visualized by silver staining and purified with *Montage* (Multiscreen PCR, Millipore Corporation, USA). We performed sequence reaction products on each strand by means of the ABI Prism dRhodamine Terminator cycle sequencing reaction kit (Applied Biosystems). DNA products were then purified by ethanol precipitation and sequence reaction products analyzed on the ABI Prism 3100 automatic sequencer (Applied Biosystems). We omitted population variation at the hypervariable sites (mainly related to the cytosine homopolymeric track around 310 and the CA-dinucleotide repeat around positions 522) from inter-population comparisons and phylogeographic analyses. We have used the same primers for amplification and sequencing described in [4].

Sequences were edited using the numbering system of the revised Cambridge Reference Sequence [5]. Most of the sequences could be read from np 16042–16569 and 21–550; for convenience, we will refer to these as HVS-I and HVS-II, although these sequence ranges encompass more than the canonical ranges of these control-region segments.

### Quality checking

Problems with the quality of mtDNA data in forensic, clinical, and population genetic studies are unfortunately rather common; see, for instance, [6], [7], [8], [9], [10], [11]. In order to minimize the effects of potential laboratory and documentation errors, the data were read separately by two independent persons in the light of the known phylogeny. We checked phylogenetic inconsistencies by hand with special attention to private or unusual variants (e.g. rare transitions or indels). In some cases, we confirmed the sequences by repeated extraction and sequencing. In addition, to detect potential “phantom mutations” [7], we also checked the data using the computer program SPECTRA ([7], available at http://www.stats.gla.ac.uk/~vincent/fingerprint/index.html).

### Statistical analysis and population comparison

Haplogroup nomenclature follows the most recently updated versions of the Native American phylogeny given in [12], [13], [14]. Diversity indices of HVS-I sequences (haplotype diversity, nucleotide diversity, average number of pairwise differences) were calculated using Arlequin 3.0 software [15]. Nucleotide and sequence diversity was computed as in the manner of Nei [16].

We estimated median-joining networks of HVS-I sequences using the Network 4.1.1.2 software [17], [18]. Coalescence times were calculated using the ρ statistic [19], [20] with an HVS-I mutation rate of one transition per 18,845 years applied for the sequence range 16090-16365 using the most recent estimates provided by Soares et al. [21].

An mtDNA database of Native American populations was compiled for population comparisons: (i) from North America: Aleuts [22], Athapaskans, Inupiaq, Yakima [23], Chukchi and Siberian Eskimos [24], Bella Coola and Haida [25], Nuu-Chah-Nulth [26], Cheyenne [27], North Native Americans [various ethnic groups; 28], [29], [30], [31], [32], [33], Apache and Navajo [34], (ii) from Meso-America: Pima [27], Maya [33], Huetar [35], Kuna [36], Ngöbe [37], Quiché [38], Emberá and Wounan [39], Mexico [40], Central Native Americans [various ethnic groups] [28], El Salvador (present study), and (iii) from South America: Native Brazilians and Araucanians or Chileans [33], Equador [41], Embera and Gavião [42], Amazonas [43], Ayoreo [44], Chilean Mapuche and Pehueche, Yaghan [45], Argentinian Mapuche [46], Cayapas [41], Xavante, Zoró, and Gavião [42], Yanomami [47], [48], South Native Americans [various ethinic groups] [29], Tuacuarembó [49], Uruguay [50], Guahibo [51], Colombia [33], [52], Yuracaré, Trinitario, Movima, and Ignaciano [53], and 105 from Arequipa, Tayacaja and San Martin in Peru [54]. We also included the data collected from several studies of ancient DNA [55], [56], [57], [58], [59], [60], [61]. In addition, other datasets were additionally used for haplotype matching comparisons [34], [62], [63], [64], [65], [66]. In total, 3,843 mtDNAs profiles (mainly HVS-I segments) were compiled for comparison with our sample from El Salvador. Those population samples consisting of less than 15 individuals were only used for haplotype matching between populations. For comparison purposes the common reading frame 16090–16365 of the HVS-I was used.

### Admixture analysis

We took two different approaches to carry out an admixture analysis.

The first model was applied as described in [67] although, instead of using haplogroup frequencies as variables, we used the frequencies of the shared haplotypes (matching haplotypes) between the source populations (North and South America) and El Salvador. The number of mtDNAs within each matching haplotype in El Salvador (*n_i_*: 1≤*i*≤*C*, the number of different matching haplotypes in the sample) was assumed to be a draw from a multinomial distribution with parameters 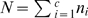 and 
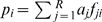
 (1≤*i*≤*C*), where *R* is the number of source regions in America, *f_ji_* is the frequency of the *i*th cluster in the *j*th source region (assumed to be known), and α*_j_* are the admixture coefficients. This model describes samples from an urn containing *C* different kinds of ball, where the urn has been created by mixing together *R* other urns in proportions given by the admixture coefficients. We chose to analyze this model in a Bayesian framework, which meant that we had to explore the distribution of the admixture coefficients, given the data. The prior distribution of the admixture coefficients was taken to be uninformative—namely, uniform on 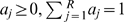. The posterior distribution of the {*α_j_*} was explored with the Metropolis-Hastings algorithm, using a simple proposal, and was summarized by the posterior mean of each a*_j_* and its root-mean-square deviation about the mean. To assess model fit, we examined plots of standardized residuals.

The second admixed model was applied as described [68]. The probability of origin of each of the sub-continental region was computed as 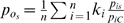 where, *n* is the number of El Salvador sequences with matches (≥1) in the whole continental dataset; *k_i_*, the number of times the sequence *i* is found in El Salvador; *p_is_*, the frequency of the sequence *i* in the sub-continental region dataset; and *p_ic_*, the frequency of the sequence *i* in whole continental dataset.

### Founder analysis

The time to the most recent common ancestor (TMRCA) of haplogroup A2 in the phylogeny was estimated as described [19], [20].

In order to carry out a founder analysis [19], [69], we made some simplifying assumptions about the founding of El Salvador. We assumed (i) a single migration to El Salvador and (ii) that North America was the unique source population. Founder sequences were inferred as matches with samples from North America. An estimate of the time of the migration event was determined by averaging diversity over the clusters derived from each founder in El Salvador, as follows. Suppose there are *r* founder clusters. Let, *ρ_i_* be the *ρ* value (average distance of the haplotypes of a clade from the respective root [19], [20]) for the *i^t^* ^h^ founder cluster, *σ_i_* be its estimated standard error [20] and *n_i_* be the number of sampled individuals in that cluster. Define,
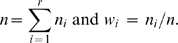
Then,
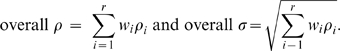
Values of *ρ* and σ were converted to age using the most recent mutation rate available for the HVS-I segment of 1 transition per 18,845 years (in the sequence range 16090–16365).

## Results

### Summary statistics

We observed a total of 55 different HVS-I, 40 different HVS-II, and 76 different combined HVSI/II mtDNA haplotypes in El Salvador (*N* = 90). Some HVS-I profiles are quite common, such as C16111T–T16223C–C16290T–G16319A–T16362T, appearing in 12 mtDNAs, and its one step-mutation ‘neighbour’ haplotype (16519 on top) appearing 12 times.

As shown in Table 1, El Salvador shows haplotype and nucleotide diversity values slightly lower than those observed in the continental North, South, and other Meso American populations, which is in part due to the fact that there is virtually only one Native American haplogroup (A2) represented in El Salvador sample. Note that these comparisons have to beviewed with care because the terms “North”, “South” and “Meso-American” refer to groups of population samples of different nature; some are Native American groups that have passed through severe prehistoric bottlenecks while others are at different levels of admixture with *e.g.* Europeans and Africans.

**Table 1:** Native American population mtDNA database considering the sequence range 16090–16365.

	n	H	D	π	M
El Salvador	90	49	0.917±0.025	0.013±0.002	3.5
North America	1215	243	0.950±0.003	0.020±0.000	5.1
Meso America	394	142	0.968±0.004	0.023±0.012	6.2
South America	1144	265	0.956±0.003	0.019±0.000	5.3

*n* = sample size; *H* = number of different haplotypes; *D* = haplotype diversity; *π* = nucleotide diversity; *M* = average number of nucleotide differences.

### Phylogeography of Salvadorian Native American mtDNAs

Table 2 shows the full list of control region profiles from El Salvador and their haplogroup allocation. Frequencies of the typical Native American haplogroups A2, B2, and C1 are ∼91%, ∼2%, and ∼2%, respectively.

**Table 2:** MtDNA haplotypes in El Salvador.

ID#	HVS-I (minus 16000)	HVS-I reading range (minus 16000)	HVS-II	HVS-II reading range	HG
1	42 111 223 244 290 319 362 519	024-569	64 73 146 152 153 154 235 263 309+C 315+C 523-524del	021-540	A2
2	51 111 223 290 299 319 362	024-569	64 73 146 153 235 263 315+C 523-524del	021-589	A2
90	111 136 153 223 290 311 319 362	024-560	64 73 146 153 235 263 309+CC 315+C 523-524del	021-595	A2
10	111 136 153 223 290 319 362	024-520	64 73 146 153 235 263 309+CC 315+C 523-524del	021-590	A2
8	111 136 223 290 311 319 362	024-569	64 73 146 153 235 263 309+C 315+C 523-524del	021-560	A2
43	111 136 223 290 311 319 362	024-569	64 73 146 153 235 263 309+C 315+C 523-524del	021-600	A2
55	111 136 223 290 319 362	024-560	64 73 146 153 235 263 309+C 315+C 523-524del	021-600	A2
62	111 136 223 290 319 362	024-560	64 73 146 153 235 263 309+C 315+C 523-524del	021-540	A2
93	111 136 223 290 319 362	024-569	64 73 146 153 235 263 309+C 315+C 523-524del	021-600	A2
61	111 172 223 290 319 362 519	024-589	64 73 146 153 195 235 263 309+CC 315+C	021-590	A2
83	111 175 223 290 300 319 362	024-569	64 73 153 235 263 309+C 315+C	021-440	A2
77	111 175 290 300 319 362	024-569	64 73 146 153 235 263 309+C 315+C 523-524del	021-560	A2
35	111 181 187 223 290 304 319 362	024-564	64 73 146 153 207 235 263 309+C 315+C	021-320	A2
64	111 182C 183C 189 223 290 319 362	024-549	64 73 146 153 235 263 309+CC 315+C	021-320	A2
67	111 182C 183C 189 223 290 319 362	024-560	64 73 146 153 235 263 315+C 523-524del	021-600	A2
48	111 183C 189 223 290 319 362 381 519	024-560	64 73 146 153 235 263 309+C 315+C	021-310	A2
46	111 187 209 223 290 319 362 371 519	024-560	64 73 146 153 235 263 309+C 315+C 523-524del	021-550	A2
40	111 187 223 234 290 319 362 390 519	024-569	64 73 146 153 235 263 309+C 315+C 523-524del	021-570	A2
54	111 187 223 290 319 362	024-589	64 73 146 153 235 263 309+C 315+C	021-410	A2
6	111 187 223 290 319 362	024-569	64 73 146 153 235 263 309+C 315+C 523-524del	021-560	A2
13	111 187 223 290 319 362	024-520	64 73 146 153 235 263 309+C 315+C 523-524del	021-535	A2
17	111 187 223 290 319 362	024-530	64 73 146 153 235 263 315+C 523-524del	021-569	A2
47	111 189 223 290 319 362	024-530	64 73 146 153 235 263 309+C 315+C 523-524del	021-550	A2
89	111 189 223 274 290 319 362	024-540	64 73 146 153 235 263 309+CC 315+C	021-510	A2
87	111 189 223 290 311 319 362	024-550	64 73 146 153 235 263 292 309+C 315+C 523-524del	021-600	A2
24	111 189 223 290 319 324 362	034-569	64 73 146 153 235 263 309+CC 315+C 523-524del	021-535	A2
32	111 189 223 290 319 362	024-569	64 73 146 153 235 263 309+C 315+C 523-524del	021-535	A2
57	111 189 223 290 319 362	024-560	73 146 152 153 235 263 309+CC 315+C	021-320	A2
75	111 209 223 290 291 319 362 477	024-569	64 73 146 152 153 235 263 315+C 523-524del	021-600	A2
74	111 209 223 290 293C 319 362 519	024-569	64 73 146 153 235 263 309+CC 315+C4 523-524del	021-550	A2
28	111 209 223 290 319 362 519	024-569	64 73 146 153 235 263 309+C 315+C 523-524del	021-600	A2
31	111 209 223 290 319 362 519	024-569	64 73 146 153 235 263 309+C 315+C 523-524del	021-595	A2
99	111 223 234 290 319 362 519	024-569	64 73 146 153 235 263 309+C 315+C 356+C 523-524del	021-600	A2
21	111 223 243 290 299 319 362	024-569	64 73 146 153 235 263 309+C 315+C 523-524del	021-600	A2
7	111 223 290 299 319 362	024-569	64 73 146 153 235 263 309+C 315+C 523-524del	021-560	A2
37	111 223 290 299 319 362	024-560	64 73 146 153 235 263 309+C 315+C 523-524del	021-535	A2
44	111 223 290 299 319 362	024-569	64 73 146 153 235 263 309+C 315+C 523-524del	021-600	A2
49	111 223 290 299 319 362	024-520	64 73 146 153 235 263 309+C 315+C 523-524del	021-570	A2
82	111 223 290 300 319 362	024-569	64 73 146 153 235 263 309+C 315+C 523-524del	021-600	A2
23	111 223 290 311 319 360 362	024-569	146 153 235 263 309+CC 315+C 523-524del	021-539	A2
26	111 223 290 311 319 362	024-569	64 73 146 153 235 263 309+C 315+C 523-524del	021-600	A2
4	111 223 290 311 319 362	024-569	64 73 146 153 235 263 315+C 523-524del	021-600	A2
3	111 223 290 319 360 362	024-569	143 146 152 153 204 235 263 309+CC 315+C 523-524del	021-570	A2
59	111 223 290 319 362	024-560	64 73 146 152 153 215 235 263 309+C 315+C 523-524del	021-600	A2
92	111 223 290 319 362	024-569	64 73 146 152 153 235 263 315+C	021-320	A2
38	111 223 290 319 362	024-560	64 73 146 153 235 263 309+C 315+C	021-535	A2
78	111 223 290 319 362	024-520	64 73 146 153 235 263 309+C 315+C 523-524del	021-600	A2
79	111 223 290 319 362	024-530	64 73 146 153 235 263 309+C 315+C 523-524del	021-600	A2
84	111 223 290 319 362	024-569	64 73 146 153 235 263 309+C 315+C 523-524del	021-530	A2
20	111 223 290 319 362	024-549	64 73 146 153 235 263 309+CC 315+C	021-560	A2
29	111 223 290 319 362	024-500	64 73 146 153 235 263 309+CC 315+C 523-524del	021-570	A2
36	111 223 290 319 362	024-560	64 73 153 214 235 263 315+C 523-524del	021-600	A2
63	111 223 290 319 362	024-525	73 146 150 153 235 263 315+C 523-524del	021-560	A2
68	111 223 290 319 362	024-550	n.d	–	A2
42	111 223 290 319 362 391	024-569	64 73 146 153 235 263 309+CC 315+C 523-524del	021-600	A2
103	111 223 290 319 362 518 519	024-569	64 73 146 153 235 263 315+C 523-524del	021-550	A2
91	111 223 290 319 362 519	024-560	64 73 146 150 153 174+C 235 263 309+CC 315+C 523-524del	021-320	A2
53	111 223 290 319 362 519	024-569	64 73 146 150 153 235 263 315+C 523-524del	021-600	A2
34	111 223 290 319 362 519	024-569	64 73 146 150 153 235 263 315+C 523-524del	021-600	A2
76	111 223 290 319 362 519	024-550	64 73 146 152 153 235 263 309+C 315+C	021-320	A2
27	111 223 290 319 362 519	024-569	64 73 146 152 153 235 263 309+C 315+C 523-524del	021-589	A2
14	111 223 290 319 362 519	024-569	64 73 146 153 235 263 309+C 315+C 523-524del	021-600	A2
51	111 223 290 319 362 519	024-569	64 73 146 153 235 263 309+C 315+C 523-524del	021-600	A2
86	111 223 290 319 362 519	021-569	64 73 146 153 235 263 309+C 315+C 523-524del	021-600	A2
88	111 223 290 319 362 519	024-569	64 73 146 153 235 263 309+C 315+C 523-524del	021-600	A2
11	111 223 290 319 362 519	024-569	64 73 146 153 235 263 315+C 523-524del	021-570	A2
9	111 223 290 319 362 519	024-569	73 146 152 153 197 235 263 309+C 315+C 523-524del	021-590	A2
15	111 223 290 319 362 519	024-569	73 146 153 235 263 309+C 315+C 523-524del	021-600	A2
25	111 223 290 319 519	024-569	64 73 146 153 235 263 309+C 315+C 523-524del	021-544	A2
97	111 261 290 319 362 519	024-550	73 146 152 153 235 263 315+C 523-524del	021-600	A2
45	111 290 311 319 362 391	024-569	64 73 146 153 235 263 315+C 523-524del	021-590	A2
50	111 290 311 319 362 391	024-569	64 73 146 153 235 263 315+C 523-524del	021-589	A2
19	111 290 319 362 391	024-569	64 73 146 153 235 263 309+C 315+C 523-524del	021-600	A2
58	153 223 240 290 319 362	024-560	64 73 146 153 235 263 309+C 315+C 523-524del	021-590	A2
18	189 223 290 319 362	024-569	64 73 146 153 235 263 309+C 315+C 523-524del	021-585	A2
56	223 290 311 319 362	024-560	64 73 146 153 235 263 309+C 315+C 523-524del	021-530	A2
5	223 290 316 319 362	024-569	64 73 146 153 182 235 263 309+C 315+C 523-524del	021-560	A2
60	223 290 319 352 362	024-560	64 73 146 153 182 235 263 309+C 315+C 523-524del	021-589	A2
70	223 290 319 362	024-545	64 73 146 153 235 263 315+C 523-524del	021-600	A2
100	223 290 319 362 519	024-569	64 73 146 153 235 263 309+C 315+C 523-524del	021-600	A2
16	92 111 189 223 290 319 362 519	024-569	64 73 146 153 235 263 309+C 315+C 523-524del	021-570	A2
81	93 111 136 223 290 319 324 362	024-569	73 146 235 263 309+C 315+C 523-524del	021-580	A2
33	129 183C 189 217 269 519	024-569	73 146 152 195 234 263 309+CC 315+C 499	021-570	B2
22	183C 189 217 519	024-569	73 263 309+C 315+C 499	021-570	B2
41	183C 189 223 256 298 325 327	024-560	73 249del 263 290-291del 309+C 315+C 489 523-524del	021-555	C1
39	189 223 298 325 327 362 519	024-560	73 195 249del 263 290-291del 315+C 489	021-580	C1
12	519	024-569	153 263 315+C 523-524del	021-600	H?
52	129 148 168 172 187 188G 189 223 230 278 293 311 320	024-560	93 95C 185 189 236 247 263 315+C 523-524del	021-560	L0a1a
30	126 271 294 296 304 519	024-569	73 263 315+C	021-580	T2
96	51 129C 189 362	024-540	73 152 217 263 309+C 315+C 340 508	021-600	U2e

HG = haplogroup.

n.d. = non determined.

**Figure 1:**
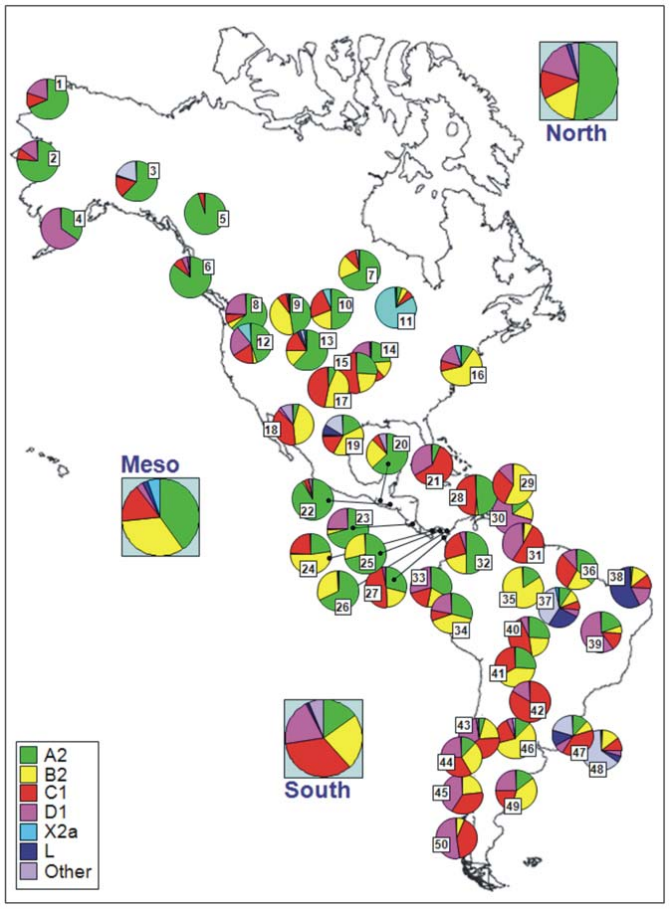
Haplogroup patterns in America. Codes for populations are as follow: North America: 1 = Chukchy, 2 = Eskimos [24]; 3 = Inuit (collected from the HvrBase database [84]; 4 = Aleuts [22]; 5 = Athapaskan [23]; 6 = Haida [25]; 7 = Apache [85], 8 = Bella Coola [25]; 9 = Navajo [85]; 10 = Sioux, 11 = Chippewa [66], 12 = Nuu-Chah-Nult [26]; 13 = Cheyenne [66]; 14 = Muskogean populations [31]; 15 = Cheyenne-Arapaho [30]; 16 = Yakima [23]; 17 = Stillwell Cherokee [30]; Meso-America: 18 = Pima [27]; 19 = Mexico [40]; 20 = Quiche [38]; 21 = Cuba [38]; 22 = El Salvador (present study); 23 = Huetar [35]; 24 = Emberá [39]; 25 = Kuna [36]; 26 = Ngöbé [37]; 27 = Wounan [37]; South America: 28 = Guahibo [51]; 29 = Yanomamo from Venezuela [48]; 30 = Gaviao [42]; 31 = Yanomamo from Venezuela and Brazil [86]; 32 = Colombia [52]; 33 = Ecuador (general population), 34 = Cayapa [41]; 35 = Xavante [42]; 36 = North Brazil [43]; 37 = Brazil [64]; 38 = Curiau [60]; 39 = Zoró [42]; 40 = Ignaciano, 41 = Yuracare [53]; 42 = Ayoreo [44]; 43 = Araucarians [33]; 44 = Pehuenche, 45 = Mapuche from Chile [45]; 46 = Coyas [4]; 47 = Tacuarembó [49]; 48 = Uruguay [50]; 49 = Mapuches from Argentina [46]; 50 = Yaghan [59].

Figure 1 shows the frequency distribution of the main mtDNA American haplogroups in Native American populations. Although haplogroup A2 is at high frequencies in Meso America, El Salvador is particularly distinct from the other populations by its extremely high A2 haplogroup frequency. Note also that there exists substantial heterogeneity of haplogroup frequency patterns in America (even between neighbouring populations).

**Figure 2:**
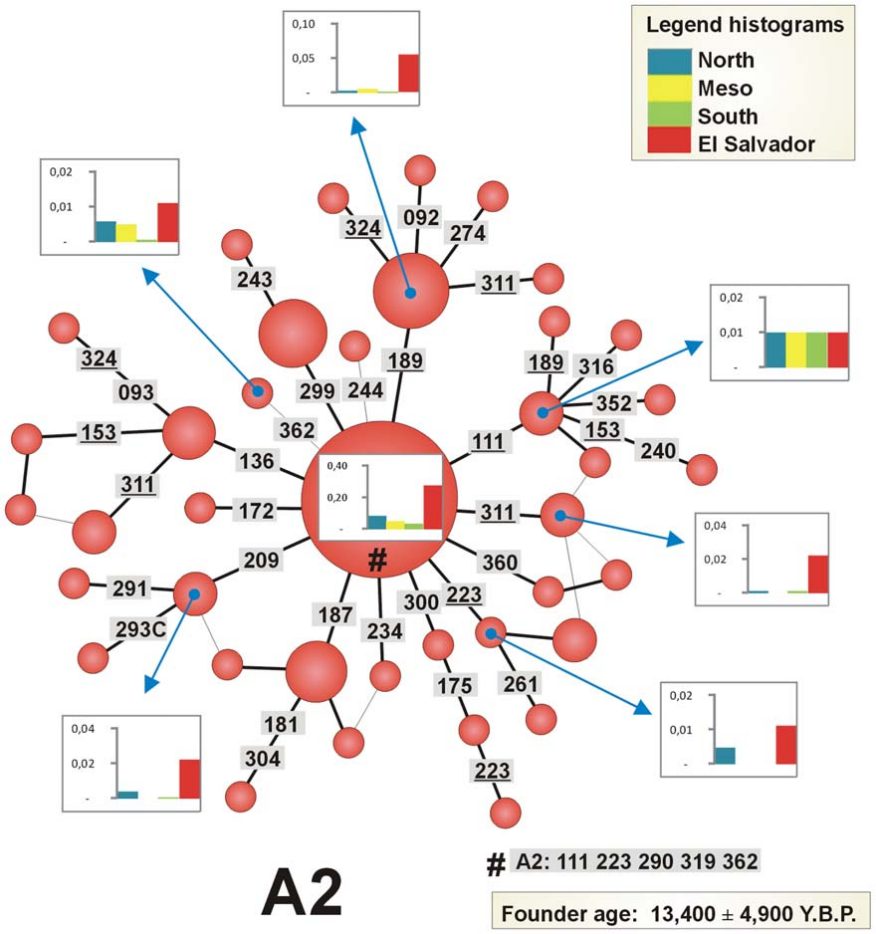
Phylogenetic network of haplogroup A2 mtDNA sequences from El Salvador. Only the variation contained in the HVS-I segment (range 16090–16365) was used. Positions are referred to the rCRS minus 16000; a transversion is specifically indicated as a suffix. The areas of the circles are proportional to the number of individuals bearing the corresponding haplotype in the El Salvador sample. The histograms indicate the frequency of the El Salvadorian mtDNA founder clusters in other continental regions (North, Meso, and South America). To compute the TMRCA and founder ages, we resolved the network to a tree (shown by the bold connections between haplotypes), using the positional mutation rates reported in [21]. Resolved parallel mutations are shown underlined. The ancestral haplotype is indicated by a hash.

The phylogeny of A2 in El Salvador is clearly star-like (Figure 2); its root is, identified by the diagnostic sites C16111T–T16223C–C16290T–G16319A–T16362C in HVS-I, and C64T–A73G–T146C–A153G –A235G–A263G–315+C in HVS-II. There are no very solid diagnostic sites in the control region that would allow us to classify A2 sub-lineages from El Salvador [12], [14]. Moreover, several control-region variants regarded as haplogroup diagnostic, such as C64T and A153G, show reversions: complete genome sequence data confirm the existence of multiple back and parallel mutations within haplogroup A2 [12], [14]. Although many of them are well-known hotspots (e.g. T146C), others such as position 64, seem to behave as hotspots only within A2 (see e.g. [12]). Other Native American lineages, like D1, D4h3 and X2a [13] are absent from our sample from El Salvador.

The sub-clade of A2 carrying C16360T is particularly prevalent in Meso America, especially in the Huetar (12 matches; ∼44% of the Huetar sample) from Costa Rica [35] and the Ngöbé (three matches; ∼7% of the Ngöbe sample) from Panama [37]; in El Salvador this variant was also present in two individuals. The haplotype C16111T–C16187T–T16223C–C16290T–G16319A–T16362C is virtually only shared with the Ngöbe (19 matches that make up ∼41% of the Ngöbe sample) but was also detected in one Uruguayan [50]. El Salvador shares a higher number of haplotypes with North America (19), followed by Meso-America (10), and then South America (8); note however that the database for Meso-America (*n* = 395) is of a much lower sample size than the one from North (*n* = 2,010) and South (*n* = 1,596). These results roughly indicate a clear imprint of North in Meso-America and also the existence of lineages that are mainly concentrated in Meso-America (probably due to the fact that these were founders in the region and experienced posterior expansion); in some instances, some of these South mtDNAs could have been carried from Meso-America in some wave of migration towards the South (such as the one indicated above observed in Uruguay, or *e.g.* C16111T–T16189C–T16223C–C16290T–16311–G16319A–T16362C, which was also found in three Brazilians [64]).

We found only four Native American mtDNAs not belonging to haplogroup A2: two haplogroup B2 and two haplogroup C1 mtDNAs. We did not find any exact match amongst published data for the B2 sequence #33 that carries the distinctive variant A16269G. The haplogroup C1 sequence #41 carries C16256T; this uncommon variant within haplogroup C1 has been also observed in the Yanomama from Venezuela [47] and the Zoró from Brazil [42]. Haplotype C1 #39 was only observed in one Brazilian [43] and one Guahibo from Venezuela [51], but also in two ancient Taino samples from the Caribbean [57].

### Non-Native American haplotypes in El Salvador

Signals of a European contribution to our sample from El Salvador are limited to three haplotypes (see Table 1): haplotype #96 belongs to haplogroup U2e, with exact matches in several West European locations (e.g. Northwest Spain and Portugal [70]); in Madeira [71], etc. Haplotype #12 can be assigned most plausibly to haplogroup H, while #30 probably belongs to haplogroup T2; this sequence curiously matches published sequences only observed in Portugal and Brazil [64], [72] but also a single hit in Poland [73].

We detected only one sequence belonging to a typical sub-Saharan haplogroup in El Salvador. It belongs to L0a1a, a sub-clade highly prevalent in southeast Africa [67], [74], [75], where we find exact matches in HVS-I and HVS-II. Exact matches are also found in, for example, the Atlantic African southwest coast, in Cabinda [76], and in the Tongas [77]. Although it is not possible to determine with precision the African origin of this haplotype, southeast Africa (Mozambique) is probably the best candidate population source.

### Admixture analysis

The admixture analysis carried out as in [67] indicated that North America accounts for ∼92% of the lineages in El Salvador, the remaining ∼8% coming from South America. The method described in [68] indicated that North America contributed to El Salvador ∼76% of the mtDNA lineages, in contrast to the ∼24% coming from South America.

### Founder analysis

We inferred seven founders in our Salvadorian sample, all present in North American populations. Some sequence matches were not considered founders because they were detected only in Mexico and not in North American populations; they are more likely the result of recent gene flow between El Salvador and neighbouring populations. Some other potential founders were also rejected because they were present mostly as singletons analyzed in North American laboratories but belonging to e.g. ‘Hispanic’ or other sample populations without information about their ethnic affiliation. All the founders are indicated in Figure 1. The founder age of haplogroup A2 in El Salvador was estimated as 13,400±5,200 Y.B.P.

## Discussion

El Salvador is the smallest Latin American republic and also the most densely populated. Although historically El Salvador has been home to a culturally diverse mix of peoples, including Native Americans, Africans, and west Europeans, by the 1980s the population of the country was essentially considered to be homogeneous in terms of ethnicity and basic cultural identity. Virtually all Salvadorans speak Spanish, the official language, as their mother tongue, and the vast majority are generally characterized as “mestizos” (or “ladinos”, a term more commonly used in Central America), popularly used to refer to those persons of mosaic geographic ancestry who follow a wide variety of indigenous and “hispanic” customs and habits that over the centuries have come to constitute Spanish-American cultural patterns. In the late 1980s, the ethnic composition of the population was estimated as 89% “mestizo”, 10% Native American, and 1% “white” [78]. Therefore, in contrast to most other Central American countries, El Salvador no longer possessed an ethnically or linguistically distinct Native American population, although persons of native-like ethnicity or cultural heritage still lived in the western parts of the country. Similarly, there was no ethnically or culturally distinct African-American population as there is in neighbouring populations [79]. However, there is a general belief that much of the Salvadorian population in the 1980s had a predominantly Native American ancestry[1].

The results of the present study have shown that, in contrast to the cultural patterns observed in the today's El Salvador population, most of the mtDNA profiles found are typically Native American; haplogroup A2 account for ∼90% of the Salvadorian sample. Correspondingly, the impact of Europeans on the mtDNA pool of El Salvador is very low (∼2%). It seems that the Spanish conquerors and more recent European demographic influences did not contribute significantly to the today's genetic composition of El Salvador in the maternal side. This contrasts with the European Y-chromosome contribution to the El Salvador gene pool. According to [80] about one half in metropolitan areas and two thirds in rural populations of El Salvador belong to non-Native American haplogroups; for instance, the most common Y-chromosome haplogroup in Europe (namely, R1b) is present in El Salvador at 24% in metropolitan areas and 43% in rural regions. Concomitantly, the Native American Y-chromosome proportion in El Salvador (represented by haplogroup Q3) is about 31–49%.

Therefore, the mtDNA and Y-chromosome variation in El Salvador displays an extreme version of a pattern that was also observed in other American populations [81], [82]: the indigenous female contribution is much higher than the indigenous male contribution.

Our results show that the impact of African-American lineages on the mtDNA pool of El Salvador was very low, as indicated by the presence of only one mtDNA of sub-Saharan origin in our sample. The scarcity of the sub-Saharan component strikingly contrasts with the situation on the Caribbean coast, where (as a consequence of the Atlantic slave trade) it is clearly predominant [67], [74], [75], [79]. The Y-chromosome variation shows a similar pattern: no lineages of African ancestry have been detected in El Salvador [80].

There are no clear signals of recent genetic drift events in the general population from El Salvador, as observed in, for instance, neighbouring but isolated Native American populations such as the Ngöbé from Panamá [37] which shows extremely reduced levels of mtDNA diversity (reflecting passage through post-conquest population bottlenecks). Haplogroup A2 is at high frequency in El Salvador (∼90% of the sample) and a high percent of the lineages (45%; computed using the sequence range 16090–16365) remain unobserved in other American populations. Admixture analysis indicates that the main mtDNA influence in El Salvador can be attributed to North America. The phylogeny of A2 is rather star-like and the founder age was 12,600±4,900 years. The shape of this phylogeny points to the existence of a prehistoric demographic expansion. Considering the most recently estimated age of A2 in the American continent as a whole of 13,400±5,200 [21] (largely determined from North American samples) as a proxy for the time of the expansion into the Americas, it can be tentatively suggested that the initial settlement of El Salvador occurred rather soon after the initial colonization of the American continent, and that El Salvador largely contains the descendants of the mtDNAs in that original pool with scarce subsequent demographic influence from other American or non-American populations. Indeed, since we have genotyped samples collected in urban areas we would expect to have an even higher prevalence of the Native American component in more isolated groups from the country, as is in fact observed on the Y-chromosome side where the Native American component is higher in rural than in metropolitan areas [80].

In contrast to the high impact of the Atlantic slave trade on the Central American Caribbean coast [79], the Pacific side (at least for El Salvador) appears to have preserved its Native American mtDNA heritage intact to the present day. At the same time, this study has also shown that El Salvador harbours haplogroup frequency patterns quite different from other modern Native American communities. At the individual haplotype level, El Salvador shows numerous mtDNAs that have never been observed in other American regions, even within Central America. These features provide little support to those that assume (or claim) that “Hispanics” or Native American communities are sufficiently homogeneous to justify the portability of forensic databases from one country to another (e.g. SWGDAM; [34]); see [83] for a discussion.
